# The first complete mitochondrial genome of wild soybean (*Glycine soja*)

**DOI:** 10.1080/23802359.2018.1467228

**Published:** 2018-04-25

**Authors:** Sajjad Asaf, Abdul Latif Khan, Ahmed Al-Harrasi, Tae Han Kim, In-Jung Lee

**Affiliations:** a Natural and Medical Science Research Center, University of Nizwa, Nizwa, Oman;; b School of agriculture civil and bio-industrial machinery engineering, Kyungpook National University, Daegue, Korea;; c Crop Physiology Laboratory, School of Applied Biosciences, Kyungpook National University, Daegu, Republic of Korea;; d Research Institute for Dok-do and Ulleung-do Island, Kyungpook National University, Daegu, Republic of Korea

**Keywords:** Mitochondrial genome, wild soybean, phylogenetic analysis, Illumina sequencing

## Abstract

*Glycine soja* (wild soybean) is known as the wild progenitor of cultivated soybean (*G*. *max*), representing a valuable genetic resource for soybean breeding programmers. In this study, we determined the complete mitochondrial genome of *G. soja*. The results revealed that *G. soja* mt genome is 402,545 bp with 45% GC contents. Furthermore, based on entire mitochondrial genome the evolutionary relationship and phylogenetic analysis revealed that *G*. *soja* closely related to *G. max*.

The mitochondrion is a membrane-enclosed organelle discovered in eukaryotic cells (Henze and Martin 2003). Plant mitochondrial DNA (mtDNAs) are a major resource for evolutionary studies, because coding regions evolve slowly, in contrast to the flexible non-coding DNA. Therefore, the structural evolution and plasticity of plant mtDNAs make them powerful model for exploring the forces that affect their divergence and recombination. Soybean (*Glycine)* is standout amongst the essential worldwide crops, grown for vegetable oil and protein. Soybean nuclear and chloroplast genomes had been published (Saski et al. 2005; Schmutz et al. 2010; Asaf et al. 2017), significantly increasing our expertise of soybean biology. The arrival of excessive-throughput sequencing technology has facilitated rapid developments in the field of genomics, especially in mitogenome genetics. In the present work, the complete mitochondrial genome of wild soybean *G. soja* was sequenced (GenBank accession number: MF955859) with the aim to know its phylogenetic position on the basis of entire mitogenomes.

The *G*. *soja* (accession KLG90379), seeds were received from the National GeneBank of the Rural Development Administration of the Republic of Korea. Mitochondrial DNA was extracted from young leaves using the method described previously (Asaf et al. 2016; Wu 2016). Approximately eighty million raw Illumina reads were demultiplexed and trimmed. The raw reads were filtered and then assembled *de novo* into contigs using Geneious Pro v11.1 (http://geneious.com). BLAST searches were conducted on all of the contigs using the NCBI database (http://www.ncbi.nlm.nih.gov/) for the annotation of mitochondrial sequences. To identify tRNAs IN the genome tRNA scan-SE software (http://lowelab.ucsc.edu/tRNAscan-SE/) was used. The complete mitochondrial genome was used to infer its phylogenetic position using Neighbour-Joining (Saitou and Nei 1987) tree with 1000 bootstrap replications (Felsenstein 1985).

The complete mt genome of *G. soja* was 402,545 bp in size, encoded 111 genes, including 19 transfer RNA (trn), 3 ribosomal RNA (rrn) genes and 36 protein coding genes (eight subunits of NADH dehydrogenase, 3 subunits of cytochrome c oxidase and seven subunits of ATP synthase) (Figure 1). *Glycine soja* mt genome is 13 bp less than previously reported *G. max* mt genome. Among these genes, *atp1* and *atp 6* genes are present in three and two copies, respectively. Furthermore, Like *G. max* mitochondrial genome *G. soja* also has 52 ORFs in it mitochondrial genome (Chang et al. 2013). Similarly, the phylogenetic analysis revealed that mt genome of *G. soja* is closely related to *G. max* (Figure 1). This study will help to understand the evolution of *G. soja* mitochondrial genome within the Family Fabaceae.

**Figure 1.**
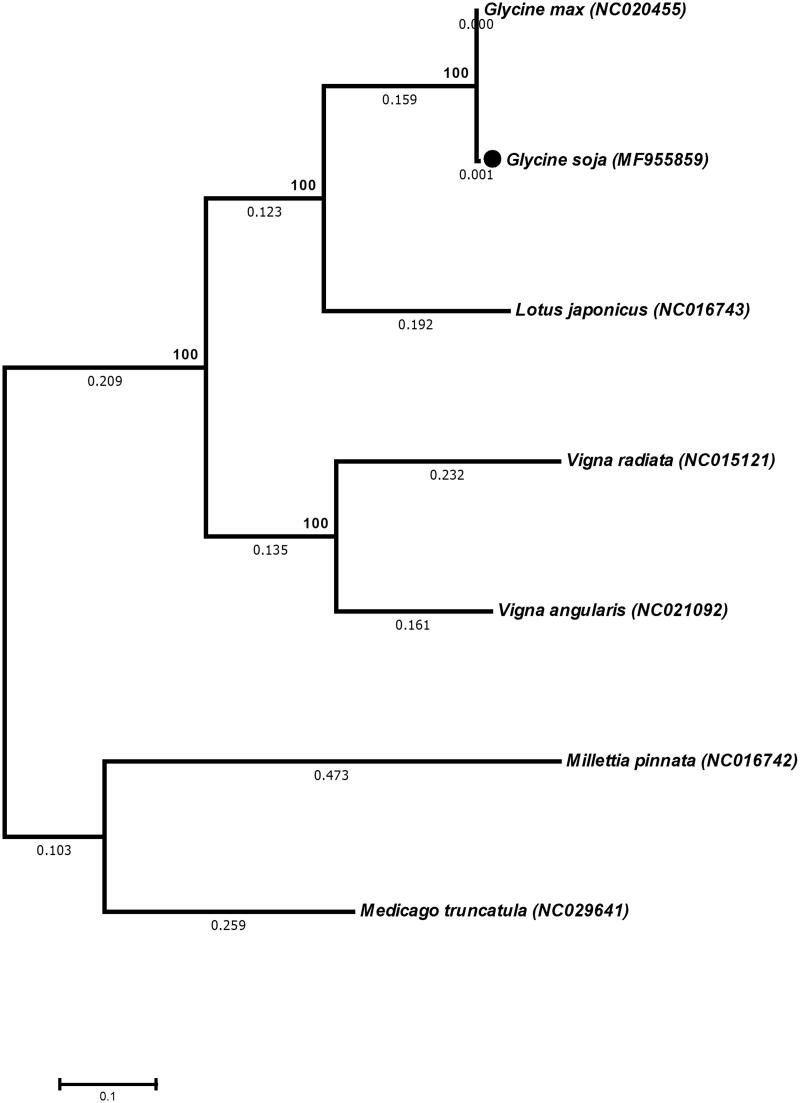
Phylogeny of the *G. soja* mitochondrial genome with six other related species form family Fabaceae. The phylogenetic tree was inferred using the neighbour-joining method based on entire mitochondrial genomes of these species.
